# Causes and Risk Factors of Repeated Hospitalization among Patients with Diabetic Retinopathy

**DOI:** 10.1155/2022/4663221

**Published:** 2022-05-28

**Authors:** Yu Xiao, Yingying Liang, Zhanjie Lin, Huiqian Kong, Zijing Du, Yunyan Hu, Shuyi Ouyang

**Affiliations:** ^1^Guangdong Eye Institute, Department of Ophthalmology, Guangdong Provincial People's Hospital, Guangdong Academy of Medical Sciences, Guangzhou, China; ^2^The Second School of Clinical Medicine, Southern Medical University, Guangzhou, China; ^3^Graduate School, Shantou University Medical College, Shantou, China

## Abstract

**Purpose:**

To identify the causes and risk factors of repeated hospitalization among patients with diabetic retinopathy (DR).

**Methods:**

Our study retrospectively examined the data of DR patients who were readmitted for treatments to the Department of Ophthalmology, Guangdong Provincial People's Hospital between January 2012 and July 2021. We first analyzed the main causes of repeated admissions and then divided the patients into three groups according to the times of readmissions. Ordinal logistic regression was performed to determine the impact of patients' demographic and clinical characteristics. Moreover, comparisons of the length of stay and the hospitalization cost of DR patients with repeated admission causes were conducted.

**Results:**

Among 2592 hospital discharges of 827 patients who experienced at least two hospitalizations, the major causes of repeated hospitalization were macular edema (30.83%), vitreous hemorrhage (29.09%), cataract (22.76%), proliferative membrane formation (6.91%), silicone oil removal (4.71%), retinal detachment (4.44%), and glaucoma (4.17%). The results of ordinal logistic regression showed that younger patients with medical insurance and local residence have a higher risk of repeated hospitalization (*p* < 0.05). Furthermore, patients readmitted for vitreous hemorrhage, proliferative membrane formation, and retinal detachment experienced longer length of hospital stay and higher hospitalization cost (*p* < 0.001).

**Conclusions:**

Multiple causes and risk factors contribute to repeated hospitalization, imposing a substantial physical and economic burden on DR patients. A better understanding of these causes and risk factors of readmission may lead to lowering such risks and alleviating patients' burden.

## 1. Introduction

Diabetic retinopathy (DR) is a common microvascular complication of diabetes mellitus. Patients with any type of diabetes are at the risk of developing DR, and it was found that after 20 years of living with diabetes, regardless of the efficacy of blood sugar management, almost all patients with type 1 diabetes and more than 60% of those with type 2 diabetes would have DR [1]. Furthermore, DR is considered the leading cause of vision loss and global blindness in working-age adults [2]. Despite statistical variations among different researches, a high prevalence of DR (up to 43.1%) was reported in diabetic population in China, contributing to the largest DR population in the world [3–6].

DR not only threatens the vision and life quality of patients, but also brings heavy healthcare and economic burden to their family and society. With worldwide population aging and prolonged life expectancy, the prevalence and burden of DR will continue to escalate [7]. As a progressive disease, DR can be divided into two stages according to its pathophysiology and corresponding severity: nonproliferative and proliferative. DR can be asymptomatic in its early, nonproliferative stage, which, without early detection and timely treatment, will furtively progress to proliferative DR, causing severe visual impairment [8]. Hence, measures should be taken to delay DR progression and therefore reduce the times of readmission, such as disease prevention and regular screening in the target population, as well as early intervention and patient education among DR patients in order to raise their awareness and compliance [9].

Hospital readmission is an undesirable outcome that may be preventable in DR management [10]. A thorough understanding of the causes and risk factors of repeated admissions is needed to identify high-risk individuals and reduce the readmission rate. Although current evidence has already indicated that long duration of diabetes, poor glycemic control, and high blood pressure are the risk factors of DR progression, the impacts of demographic and clinical characteristics on repeated admissions of DR patients remain unclear [11].

This study aimed to identify the causes and risk factors of repeated hospitalization of DR patients, as well as to compare the length of hospital stay and hospitalization cost by different causes.

## 2. Methods

### 2.1. Subjects and Data Collection

In this retrospective study, we examined the electronic medical records (EMRs) of DR patients who were readmitted to the Department of Ophthalmology, Guangdong Provincial People's Hospital (GPPH) between January 1, 2012, and July 31, 2021. Inclusion criteria was patients with the diagnosis of DR defined by an International Classification of Disease, Tenth Revision (ICD-10) code within E10-14. Patients were excluded if they did not receive treatments due to any contraindications or an undocumented reason (no specific reason noted in patient record). All the treatments were performed by senior vitreoretinal specialists in accordance with the uniform operating standards of the surgical team of the Department of Ophthalmology, GPPH.

The following demographic and clinical characteristics of each patient per stay were assessed: age upon the first hospitalization, gender, residence (inside or outside the city of Guangzhou), marital status (single, married, divorced or widowed), payment type (self-pay or medical insurance), times of readmission, length of stay, hospitalization cost, and admission diagnoses including primary diagnosis and secondary diagnoses (e.g., comorbidities and systemic conditions).

Informed consent was waived since our study was based on a retrospective analysis of EMRs, with all patient information deidentified and kept anonymous. Approval of the use of these records for the analysis was provided by the Research Ethics Committee of GPPH (No.GDREC2020229H).

### 2.2. Outcome Variables

The primary outcomes were the causes and times of repeated hospitalizations. We also identified the associations between patients' demographic and clinical characteristics and the causes of repeated admissions. Furthermore, we analyzed the relationship between the causes and the length of stay, along with their association with inpatient charges.

### 2.3. Statistical Analysis

The summary of categorical variables (e.g., gender, residence, payment type, and marital status) included counts and percentages. Since no data was normally distributed with regard to all continuous variables (e.g., age, length of stay, and hospitalization charges), medians with interquartile range (IQR) in addition to mean with standard deviation (SD) values were presented.

To analyze the contributing factors to the times of hospitalization, we divided the patients into three groups. Chi-square and Fisher's exact test were performed for categorical variables, while Kruskal-Wallis rank sum test was performed for continuous ones. Variables independently associated with the times of hospitalizations at the *p* < 0.05 level on univariate analysis were retained in the ordinal logistic regression analysis. Odds ratios (OR) with a 95% confidence interval (CI) were reported for the three groups.

In addition, Mann–Whitney *U* tests were conducted to compare the length of hospital stay and inpatient fees for DR patients with different hospitalization causes and systemic diseases. A *p* value less than 0.05 was considered statistically significant for all the tests. All statistical analyses were performed using SPSS 26.0 (SPSS. Inc, Chicago, IL, USA).

## 3. Results

### 3.1. General Patient Characteristics

A total of 1623 DR patients were identified between January 1, 2012, and July 31, 2021, of which 827 patients (50.96%) experienced at least two hospital admissions. Among DR patients with multiple admissions, 436 (52.72%) were males and 391 (47.38%) were females, and the median follow-up time was 5.03 (IQR: 1.53-13.10) months. Median age of these patients upon the first hospitalization was 56 (IQR: 49-63) years. Most of DR patients were type 2 diabetes (97.58%).

With respect to residence, 308 (37.24%) of patients lived in local neighborhood, while 519 (62.76%) lived outside the city. As for marital status, 744 (89.96%) of patients were married, 24 (2.90%) were single, while 59 (7.13%) were divorced or widowed. Regarding payment type, 178 (21.52%) patients paid by themselves, while 649 (78.48%) patients had medical insurance.

### 3.2. Reasons for Repeated Hospitalization

2592 hospital discharges of 827 patients were analyzed. The major causes of repeated hospitalization were macular edema (799, 30.83%), vitreous hemorrhage (754, 29.09%), cataract (590, 22.76%), proliferative membrane formation (179, 6.91%), silicone oil removal (122, 4.71%), retinal detachment (115, 4.44%), and glaucoma (108, 4.17%). The statistic descriptions of repeated admission causes are summarized in Table 1. In addition, we analyzed the repeated admission causes of DR patients in the past 5 years, which has no statistically significant difference to that in the past decade (*p* = 0.089, Table S1 in the supplement).

### 3.3. Analysis of the Times of Hospitalizations

Among 827 patients who experienced at least two hospitalizations, the median times of hospitalizations was 3 (IQR: 2-4), with the most being 14. The times of hospitalizations of those patients were divided into the following categories: 2-3 times, 4-5 times, and >5 times. These breakpoints were established based on their clinical and statistical significance. Additionally, we analyzed the times of hospitalizations of DR patients in the past 5 years, and in the past 10 years, no statistically significant difference was found (*p* = 0.813, Table S2 in the supplement).

Table 2 illustrated the characteristics in association with the times of hospitalizations. Kruskal-Wallis rank sum test indicated that the age of patients was significantly different among three groups. Chi-square test indicated that residence (*p* = 0.003) and payment type (*p* = 0.003) were significantly different in between-group analysis. Fisher's exact test identified that marital status (*p* = 0.002) was different among those groups. However, there was no statistically significant difference between groups for gender (*p* = 0.088).

Factors that were statistically significant on univariate analysis were introduced into the ordinal logistic regression analysis (Table 3). The results showed that younger age of DR patients upon the first hospitalization was a risk factor for increasing the times of admissions (OR = 0.954; 95% CI 0.938 to 0.970; *p* < 0.001). Moreover, DR patients who lived in local neighborhood experienced more admission times compared to those who lived outside the city (OR = 1.948; 95% CI 1.370 to 2.773; *p* < 0.001). Besides, individuals with medical insurance were at higher risk of repeated hospitalization than those who paid the inpatient cost by themselves (OR = 1.797; 95% CI 1.130 to 2.858; *p* = 0.013).

### 3.4. Hospitalization Causes Associated with Length of Stay and Hospitalization Cost

We further analyzed the length of hospital stay and inpatient fees for DR patients within different hospitalization causes and systemic diseases.

Of 827 patients with 2592 hospital discharges, the median length of hospital stay was 1 (IQR: 1-4) day. The hospitalization charges per admission was 1389.65 (IQR: 955.34-2226.34) USD, while surgical fees accounted for the largest proportion (71.55%) of the total hospitalization charges, followed by medication fees (12.32%).

Regarding comorbid systemic diseases, hypertension was known in 1044 (40.28%), diabetic nephropathy in 302 (11.65%), and chronic kidney disease in 104 (4.01%) of these patients. A history of dyslipidemia was present in 70 (2.70%) of these patients, while 50 (1.93%) and 43 (1.66%) of the patients had the history of stroke and coronary heart disease, respectively.

As summarized in Table 4, we found that length of stay increased in patients with admission causes of proliferative membrane formation, vitreous hemorrhage, retinal detachment, and glaucoma. Meanwhile, patients with admission causes of macular edema and cataract experienced shorter length of stay.

Length of hospital stay was also found to be significantly longer among the DR patients with comorbid systemic diseases (including hypertension, diabetic nephropathy, chronic kidney disease, and dyslipidemia), compared to those without.

As for hospitalization cost, the nonparametric tests showed that DR patients with admission diagnosis of proliferative membrane formation, retinal detachment, and vitreous hemorrhage required higher hospitalization costs, compared to those with macular edema, cataract, glaucoma, and silicone oil removal (Table 5).

Concerning hospitalization cost between those with and without systemic diseases, the nonparametric tests demonstrated significant differences between the two groups. We found that the inpatient fees of patients with comorbid systemic diseases (including hypertension, diabetic nephropathy, chronic kidney disease, and dyslipidemia) were much higher than those without.

## 4. Discussion

Nowadays, DR poses a major public health burden to Chinese society. This study contributes to the limited number of literature on the investigation of repeated admissions in DR patients. In this study, we first determined the main causes as well as the risk factors of repeated hospitalization. Then, we investigated the association between the length of hospital stay, inpatient charges, and readmission diagnosis in each stay.

The primary diagnosis of readmissions in DR patients were macular edema (30.83%), vitreous hemorrhage (29.09%), cataract (22.76%), proliferative membrane formation (6.91%), silicone oil removal (4.71%), retinal detachment (4.44%), and glaucoma (4.17%). Among these causes, patients with vitreous hemorrhage, proliferative membrane formation, and retinal detachment experienced longer length of hospital stay and higher hospitalization cost. It is known that vitreous hemorrhage, proliferative membrane formation, and retinal detachment were the most frequent and severe complications in proliferative DR, followed by the long-term treatment, high medical cost, and poor prognosis. Hence, early intervention and timely prophylactic treatment play a crucial role in preventing disease progression. However, DR could be initially asymptomatic; thus, a reasonably considerable proportion of diabetic patients might not be actively engaged in early screening and subsequent follow-ups, as they were unaware of the severity of late-stage DR and its concurrent complications. Therefore, intensive patient education is vital to raise patients' awareness and compliance with regular follow-ups throughout DR management [12]. Through thorough ophthalmic examinations, even the most subtle lesions of fundus can be detected in time, before some treatments (e.g., retinal laser photocoagulation) can be performed in the early stages of DR to delay its progression, thereby preventing the occurrence of serious complications and reducing the hospitalization times [8].

We observed that macular edema accounted for the largest proportion of hospitalization causes, along with shorter length of hospital stay and lower hospitalization charges per stay. Macular edema is the most common complication of DR in any stage, resulting in vision loss. Currently, inpatient intravitreal (e.g., anti-VEGF agents and dexamethasone implant) is an effective treatment for macular edema [13]. However, considering its high risk of recurrence, patients with macular edema were often required to take multiple injections during the course of treatment, leading to a significant increase in the times of readmission. Therefore, although the hospitalization cost per stay for patients with macular edema is relatively lower, repeated admission still increases the economic burden of patients.

In addition, up to 22.76% of DR patients were readmitted to the hospital due to cataracts, which may be explained as follows. On the one hand, diabetes is a widely observed clinical risk factor of cataract progression. Comparing to nondiabetic patients, cataracts in diabetic patients are associated with earlier age of onset and rapid progress [14]. On the other hand, cataract surgery may accelerate the progression of DR and induce macular edema [15]. Thus, clinicians are supposed to assess the risk-benefit ratio before cataract surgery and formulate the best surgical protocol to maximize patients' benefit.

We analyzed the comorbid systemic diseases and found that patients with hypertension, diabetic nephropathy, chronic kidney disease, and dyslipidemia are associated with longer length of hospital stay and higher hospitalization cost per stay. Consequently, besides maintaining stable glucose levels, it is necessary to screen for comorbid systemic diseases at the first hospitalization in DR patients and to monitor the condition of comorbidities regularly, so as to improve the efficacy of each hospital admission and to promote the life quality of patient.

According to the ordinal logistic regression analysis, younger age upon the first hospitalization was a risk factor for repeated admissions (OR = 0.954). The age upon the first hospitalization may be related to the onset age of diabetes. Previous studies have reported the correlation between young age at diabetes onset and high complications burden, and a large population study in China has shown a decreased risk of DR with advancing age after adjusting multiple risk factors [16]. Early age of onset is often associated with severe complications and rapid progression of disease [17]. Therefore, more frequent observance and proactive management of diabetic retinopathy are critical for young-onset diabetes patients.

The times of repeated admission was significantly associated with the payment type according to the ordinal logistic regression analysis (OR = 1.797). Comparing to self-paid patients, patients with medical insurance were readmitted more frequently. For patients who were repeatedly hospitalized, the median hospitalization cost reaches 1389.65 USD. Despite the increasing coverage of medical insurance in China, 21.4% of patients still paid the hospitalization bills out-of-pocket in our study. Since DR patients bear a huge economic burden along with the psychological and physical ones, medical insurance plays a crucial role in alleviating their stress and improving their compliance [18]. Therefore, it is suggested that the government and medical insurance agencies at all levels should pay more attention to optimizing medical insurance reimbursement coverage and standards and to providing the best services within the interpretation of current insurance policies.

As for residence of DR patients, the ordinal logistic regression analysis showed that the risk of repeated hospitalization of patients who lived in other places was lower than that of local residence (OR = 1.948). This may be attributed to the complicated process of seeking medical care for patients who lived in other places; hence, treatment compliance was hard to establish well. Some patients may refuse to be admitted to the hospital because of inconspicuous clinical signs, which increased the risk of disease progression. Furthermore, with the acceleration of the urbanization process and the rapid flow of population in China, the medical institutions for readmissions of these patients may change.

This is one of the first studies to evaluate the causes and risk factors of repeated admissions for DR patients. However, there were several limitations in this study. First, it was a single-center study. Since standards for hospitalization can differ between regions and countries, a multicenter study would be preferable. Besides, further study may enhance the reliability by calculating visits in other hospitals recorded in EMRs or performing short interviews through phones. Second, as a retrospective review, its inferences about causality based on these retrospective and observational data are limited, and selection bias may not be entirely ruled out. Moreover, we found it hard to determine whether the readmissions were planned or unplanned. Lastly, this study has not evaluated the effects of laboratory values on readmissions. Future prospective controlled studies are recommended to investigate laboratory values and other risk factors associated with multiple readmissions.

## 5. Conclusion

Reasons for repeated admission of DR patients varied, while factors including younger age, medical insurance reimbursement, and living in local neighborhood were identified as risk factors for repeated hospitalization. Hospital readmission among patients with DR poses a large burden on patients and healthcare systems. A better understanding of the causes and risk factors of repeated admission among DR patients may help identify high-risk patients as well as improve interventions to reduce readmission times.

## Figures and Tables

**Table 1:** Repeated admission causes of diabetic retinopathy patients.

Repeated admission causes	Cases (*n*)	Percentage (%)
Macular edema	799	30.83
Vitreous hemorrhage	754	29.09
Cataract	590	22.76
Proliferative membrane formation	179	6.91
Silicone oil removal	122	4.71
Retinal detachment	115	4.44
Glaucoma	108	4.17

**Table 2:** Comparison of characteristics in patients with different times of hospitalizations.

Variables	Times of hospitalizations			*p* value
	2-3	4-5	>5	
Age, years, median (IQR)	57 (51-64)	53 (48-60)	52 (44-57)	<0.001^a^
Gender, *n* (%)				0.088^b^
Male	315 (72.25)	79 (18.12)	42 (9.63)	
Female	300 (76.73)	69 (17.65)	22 (5.63)	
Residence, *n* (%)				0.003^b^
Inside the city	210 (68.18)	64 (20.78)	34 (11.04)	
Outside the city	405 (78.03)	84 (16.18)	30 (5.78)	
Payment type, *n* (%)				0.003^b^
Self-pay	149 (83.71)	23 (12.92)	6 (3.37)	
Medical insurance	466 (71.8)	125 (19.26)	58 (8.94)	
Marital status, *n* (%)				0.002^c^
Single	16 (66.67)	1 (4.17)	7 (29.17)	
Married	551 (74.06)	141 (18.95)	52 (6.99)	
Divorced/widowed	48 (81.36)	6 (10.17)	5 (8.47)	

^a^Kruskal-Wallis rank sum test; ^b^chi-square test; ^c^Fisher's exact test.

**Table 3:** The ordinal logistic regression of factors associated with the times of hospitalizations.

Variables	*β*	OR	95% CI		*p* value
			Lower	Upper	
Age	-0.047	0.954	0.938	0.970	<0.001
Residence					
Outside the city	Ref.				
Inside the city	0.667	1.948	1.370	2.773	<0.001
Payment type					
Self-pay	Ref.				
Medical insurance	0.586	1.797	1.130	2.858	0.013
Marital status					
Divorced/widowed	Ref.				
Single	-0.045	0.956	0.313	2.921	0.938
Married	0.277	1.319	0.665	2.614	0.428

OR: odds ratios; 95CI%: 95% confidence interval.

**Table 4:** Comparison of length of hospital stay in diabetic retinopathy patients with or without different admission causes and systemic diseases.

	With	Without	*p* value
*Admission causes*			
Proliferative membrane formation	6 (3 − 8)	1 (1 − 4)	<0.001
Vitreous hemorrhage	4 (2 − 7)	1 (1 − 3)	<0.001
Retinal detachment	4 (2 − 7)	1 (1 − 4)	<0.001
Glaucoma	4 (2 − 8)	1 (1 − 4)	<0.001
Silicone oil removal	2 (1 − 4)	1 (1 − 4)	0.016
Cataract	1 (1 − 2)	2 (1 − 5)	<0.001
Macular edema	1 (1 − 1)	3 (1 − 5.5)	<0.001
*Systemic diseases*			
Diabetic nephropathy	3 (1 − 7)	1 (1 − 4)	<0.001
Chronic kidney disease	3 (1 − 7)	1 (1 − 4)	<0.001
Dyslipidemia	3 (1 − 8)	1 (1 − 4)	<0.001
Hypertension	2 (1 − 5)	1 (1 − 4)	<0.001
Coronary heart disease	2 (1 − 4)	1 (1 − 4)	0.879
Stroke	2 (1 − 5)	1 (1 − 4)	0.327

*p* value was obtained by Mann–Whitney *U* tests.

**Table 5:** Comparison of hospitalization cost in DR patients with or without different admission causes and systemic diseases.

	With	Without	*p* value
*Admission causes*			
Proliferative membrane formation	2700.49 (2273.18 − 3449.1)	1320.56 (923.19 − 2116.53)	<0.001
Retinal detachment	2447.18 (2185.14 − 2794.36)	1345.78 (937.07 − 2163.38)	<0.001
Vitreous hemorrhage	2111.48 (1727.95 − 2640.33)	1198.87 (850.74 − 1845.2)	<0.001
Cataract	1375.44 (1155.81 − 1780.64)	1412.83 (752.52 − 2274.82)	0.001
Silicone oil removal	1288.14 (856.46 − 1637.83)	1402.71 (964.47 − 2246.06)	0.013
Glaucoma	1265.26 (679.59 − 1957.61)	1400.08 (967.23 − 2233.89)	0.019
Macular edema	933.39 (528.01 − 1195.01)	1870.08 (1195.93 − 2458.71)	<0.001
*Systemic diseases*			
Dyslipidemia	1372.96 (1340.43 − 3104.15)	2063.11 (942.23 − 2209.47)	<0.001
Chronic kidney disease	2032.43 (1146.48 − 2510.3)	1372.96 (939.99 − 2206.5)	<0.001
Diabetic nephropathy	1950.16 (1179.24 − 2578.08)	1340.94 (923.71 − 2175.21)	<0.001
Hypertension	1302.12 (1038.63 − 2412.3)	1576.62 (798.53 − 2141.5)	<0.001
Stroke	1847.58 (1124.5 − 2789.00)	1379.92 (948.29 − 2215.63)	0.003
Coronary heart disease	1380.37 (1230.02 − 2410.72)	1908.16 (947.53 − 2221.41)	0.006

*p* value was obtained by Mann–Whitney *U* tests; the unit of value : USD.

## Data Availability

The data used during the current study are available from the corresponding author on reasonable request.

## Supplementary Materials

Supplementary Materials Table S1: the readmission reasons of diabetic retinopathy patients in the past 5 years and the past 10 years. Table S2: the times of hospitalizations of diabetic retinopathy patients in the past 5 years and the past 10 years.
