# Supplementary material for: Causes and Risk Factors of Repeated Hospitalization among Patients with Diabetic Retinopathy
Source: J Diabetes Res. 2022 May 28;2022:4663221. doi: 10.1155/2022/4663221 (PMC9167072; doi:10.1155/2022/4663221)
Supplement: Supplementary Materials — Table S1: the readmission reasons of diabetic retinopathy patients in the past 5 years and the past 10 years. Table S2: the times of hospitalizations of diabetic retinopathy patients in the past 5 years and the past 10 years. [file 4663221.f1.docx]

**Supplementary Online Content**

Table S1. The readmission reasons of diabetic retinopathy patients in the past 5 years and the past 10 years

| Repeated admission causes | 2012-2021 | | 2017-2021 | |
| --- | --- | --- | --- | --- |
|  | Cases (n) | Percentage (%) | Cases (n) | Percentage (%) |
| Macular edema | 799 | 30.83 | 641 | 34.28 |
| Vitreous hemorrhage | 754 | 29.09 | 550 | 29.41 |
| Cataract | 590 | 22.76 | 363 | 19.41 |
| Proliferative membrane formation | 179 | 6.91 | 115 | 6.15 |
| Silicone oil removal | 122 | 4.71 | 92 | 4.92 |
| Retinal detachment | 115 | 4.44 | 87 | 4.65 |
| Glaucoma | 108 | 4.17 | 75 | 4.01 |

Chi-square test: χ2 =10.983, p=0.089

Table S2. The times of hospitalizations of diabetic retinopathy patients in the past 5 years and the past 10 years

| Times of hospitalizations | 2012-2021 | | 2017-2021 | |
| --- | --- | --- | --- | --- |
|  | Cases (n) | Percentage (%) | Cases (n) | Percentage (%) |
| 2-3 | 615 | 74.37% | 421 | 72.84% |
| 4-5 | 148 | 17.90% | 110 | 19.03% |
| ＞5 | 64 | 7.74% | 47 | 8.13% |

Chi-square test: χ2 =0.413, p=0.813
